# Spotlight on early-career researchers: an interview with Alexander Wyatt

**DOI:** 10.1038/s42003-019-0302-0

**Published:** 2019-02-26

## Abstract

Dr. Alexander Wyatt is a Senior Research Scientist at the Vancouver Prostate Centre and Assistant Professor at the University of British Columbia. His research uses genomics and bioinformatics to understand lethal prostate and bladder cancer and identify potential new targets for therapy. In this latest instalment of our series highlighting early-career researchers in biology, Dr. Wyatt tells us about research interests and career and about the challenges of the demanding research faculty workload. We’re sure many of our readers will appreciate Dr. Wyatt’s advice on the importance of learning to say “no”.

**Figure:**
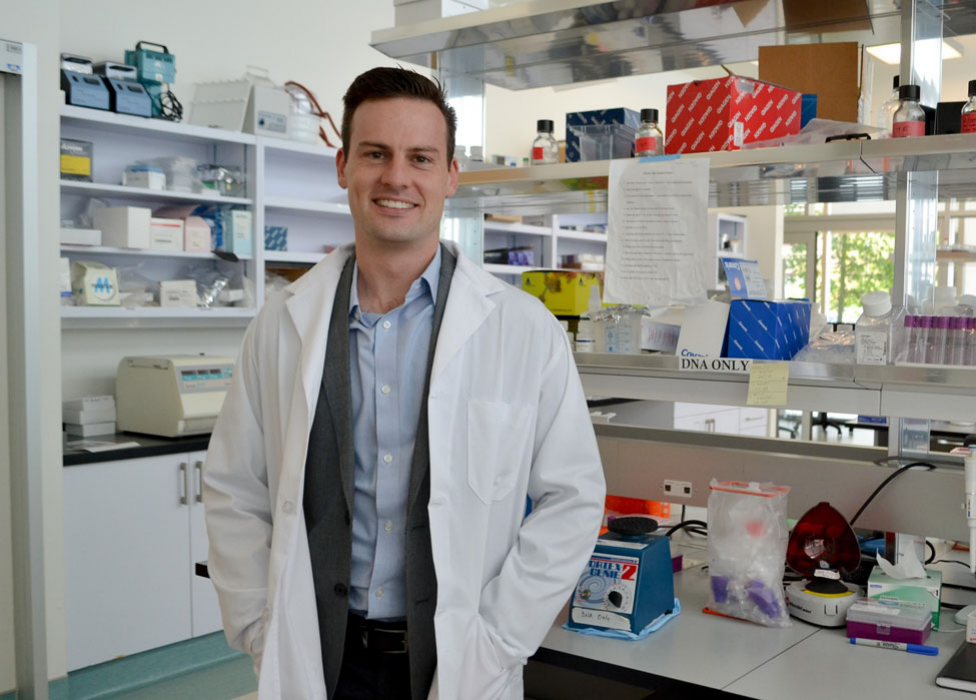
Image credit: Alexander Wyatt

Please tell us about your research interests.

I lead a small genomics and bioinformatics research group that studies metastatic prostate cancer (cancer that has spread from the prostate and colonized other tissues). Although there are lots of new life-prolonging therapies for this lethal disease, the anti-tumour response elicited by each therapy is highly variable and currently unpredictable. Our research goal is to determine the relationship between tumour genomics (e.g. the somatic alterations within each person’s cancer) and clinical responses to each therapy, including novel agents currently in early clinical trials. Of course, tumour genomics is only one part of a complex tumour-host ecosystem, and in isolation is not sufficient to explain all aspects of treatment outcomes. Nevertheless, we have identified some startling associations which we are currently testing in prospective clinical trials. If proven correct, our work will hopefully lead to better biomarkers for predicting therapy response at the individual patient level—the ‘precision oncology’ model. Perhaps the most surprising aspect of our research to those outside our field is that, in metastatic cancer, tumour genomics can often be analysed solely from a blood sample: leveraging the considerable material (DNA, RNA, cells) that cancers shed passively into circulation. This is particularly exciting as a researcher because it offers a minimally-invasive strategy (i.e. not requiring a tissue biopsy) to translate our findings into clinical practice.

What has your journey been to this point?

My PhD training at the University of Oxford was focussed on discovering genetic mutations underlying rare human congenital disorders. I was fascinated by the interplay between simple DNA mutations and complex biological consequences. It was a logical step to plan a postdoctoral fellowship in cancer, where cells can be burdened by thousands of genomic alterations. Towards the end of my PhD a close family member died of metastatic prostate cancer, and my decision was settled. I moved to Vancouver, Canada for a postdoctoral fellowship in prostate cancer genomics but had no long term career plans and remained unsure of what it actually meant to have an academic or industry career.

One year into my fellowship I was funded as a ‘Young Investigator’ by the Prostate Cancer Foundation (a non-profit organization credited for funding many of the great scientific breakthroughs in the prostate cancer research field over the past 25 years). Immediately I was part of a global community of like-minded early-career researchers. I am not really sure how it happened, perhaps it is about seeing so many brilliant peers forge their own scientific discoveries, but I found that my fascination of genomics became a singular focus to learn more and to make a difference myself. I still didn’t have a long term plan, but I knew that I wanted to lead my own research team—simply because that seemed like the best way to make things happen faster!

Towards the point in my training when I was beginning to look for independent positions, I discovered a distinguished mentor (Dr. Kim Chi) in Vancouver who provided the perfect medical oncology foil to my genomics background. Truly translational genomics research is facilitated by access to patient specimens, and with the support of Kim, I was able to plan a faculty position in Vancouver, founded upon the unfortunately steady stream of men flowing through the Vancouver urology and oncology clinics. Almost four years later I am as overworked and stressed as ever, but I have never doubted for a second that I made the correct decisions. It is an absolute privilege to have benefitted from years of higher education and public funding, and to be simultaneous trying to repay that gift while doing the very work that fascinates me most.

What are your predictions for your field in the near future?

Genomic predictive biomarkers are already proliferating in clinical research. Over the next few years it is clear that genomic sequencing of metastatic prostate cancer will become a routine part of clinical practice. I believe that tumour ‘molecular subtype’ will shortly be a new tool for urologists and oncologists to help make patient management decisions, just like patient scans or blood marker results.

Can you speak of any challenges that you have overcome?

The biggest challenge I face is the overwhelming workload, and I’m not sure that I have found a way to overcome it just yet. However, over time, each individual task seems to become less critical, and a little easier to procrastinate or complete on your own schedule rather than that of others. I think it is because when one is first launching a laboratory there is probably some truth to the feeling that one’s entire career is hinging upon each grant application, paper revision, or new student. Later on, there is some tolerability in the system; small failures seem a little more acceptable.

What advice would you give to your younger self?

A recurring piece of advice to early-career scientists, is to “just say no” to tasks and requests that are of no particular benefit for you to perform. Examples being certain projects outside the scope of your research goals, new collaborations with unclear goals, peer-review requests, committees, and so forth. However, amongst the melee of starting a laboratory, I found it very difficult to discriminate between requests that were important and requests which could be declined, especially when senior colleagues were involved. Eventually I felt comfortable trusting my gut instinct and only accepting requests that I really wanted to do, while declining those that felt like duty or where my initial reaction was negative. I also relied heavily upon my mentor to provide arm’s length advice as to which tasks were worth my time.

Scientific research is >99% failure and set-backs. It is very easy to ignore the few successes, and simply focus on the next task in hand. I did a terrible job of celebrating success as a new faculty. If I could speak to my younger self I would tell him to enjoy the wins—and not just the awarded grants and published papers, but the smaller successes: the students winning scholarships, grant applications finally being submitted, presentations that were well-received.

Finally, I would tell myself to take advantage of the troughs in between deadlines: both to recuperate but also to consolidate. It is too easy to get into the habit of relying on last-minute panic and intensity to meet deadlines!

If you could be any genome sequencing platform, which would you be and why?

Given that a lot of our work leverages post-apoptotic cell-free DNA (cfDNA) in the bloodstream of people with cancer, I could not in good conscience select anything other than a short-read sequencer! I like the adaptability of an Illumina MiSeq: it is the workhorse of so many laboratories performing a huge variety of different experiments, but has no delusions of grandeur due to its bite-sized output.

*This interview was conducted by Senior Editor, Dominique Morneau*

